# Unexpected genomic architecture in a sporadic case of C1-INH Hereditary Angioedema: the hidden heritability

**DOI:** 10.1186/s13023-025-04092-6

**Published:** 2025-11-05

**Authors:** Enrica Marchionni, Liliana Mannucci, Silvia Di Tommaso, Anna Maria Nardone, Maria Albanese, Antonio Novelli, Federica Carla Sangiuolo, Paola Triggianese, Giuseppe Novelli

**Affiliations:** 1 Medical Genetics Unit, Policlinico Tor Vergata University Hospital, Rome, Italy; 2 Translational Cytogenomics Research Unit, Bambino Gesù Children’s Hospital, IRCCS, Rome, Italy; 3 Headache Center, Neurology Unit, Policlinico Tor Vergata University Hospital, Rome, Italy; 4 Department of Systems Medicine, University of Rome Tor Vergata, Rome, Italy; 5 Department of Biomedicine and Prevention, University of Rome Tor Vergata, Rome, Italy; 6 Angioedema Reference Center, Policlinico Tor Vergata University Hospital, Rome, Italy

**Keywords:** C1-INH, HAE, SERPING1, HNPP, PMP22

## Abstract

Hereditary Angioedema (HAE) due to C1-esterase inhibitor (C1-INH) deficiency or dysfunction (C1-INH-HAE), is an autosomal dominant genetic disease characterized by recurrent cutaneous and submucosal swelling episodes most often caused by heterozygous pathogenic Single Nucleotide Variants (SNVs), small insertions or deletions (indels) or Copy Number Variants (CNVs) in the *SERPING1* gene, coding for C1-INH. Rare subtypes of HAE not associated with *SERPING1* pathogenic variants (nC1-INH-HAE) have been described. This case report aimed to characterize a 55-year-old proband, without a family history of hereditary diseases, presenting with a clinical diagnosis of C1-INH-HAE, primary Sjögren syndrome, and unexplained neuropathic symptoms. Firstly, targeted Next-Generation Sequencing and Multiple Ligation-dependent Probe Amplification (MLPA) were performed on the proband’s genomic DNA to analyze the *SERPING1* gene. Secondly, Chromosomal Microarray Analysis (CMA) and Fluorescence In Situ Hybridization analysis were performed to confirm the cytogenomics results. MLPA detected a heterozygous variant corresponding to the whole *SERPING1* gene deletion on chromosome 11, a very rare cause of C1-INH-HAE. CMA defined breakpoints and disclosed an additional pathogenic CNV on chromosome 17, involving *PMP22* gene, and causing Neuropathy, Hereditary, With Liability To Pressure Palsies disease. The clinical consequences of *SERPING1* and *PMP22* haploinsufficiency are associated with the unusual constellation of clinical symptoms, unraveling an unexpected cytogenomics architecture with two co-occurring disease-causing CNVs in a previously genetically undiagnosed patient.

To the Editor,

Hereditary angioedema (HAE) due to C1-esterase inhibitor (C1-INH) deficiency or dysfunction, is a rare autosomal dominant genetic disorder, associated with heterozygous pathogenic variants in the *SERPING1* gene (OMIM *606860), coding for C1-INH protein, leading to C1-INH-HAE (type-1 HAE and type-2 HAE; OMIM #106100). In rare cases, HAE is associated with normal levels and activity of C1-INH (nC1-INH-HAE) caused by heterozygous pathogenic variants in other genes: *F12* (OMIM *610619), *PLG* (OMIM *173350), *ANGPT1* (OMIM *601667), *KNG1* (OMIM *612358), *MYOF* (OMIM *604603), *HS3ST6* (OMIM *619210), presenting with clinical manifestations similar to C1-INH-HAE [1].

C1-INH-HAE is characterized by potentially life-threatening recurrent episodes of cutaneous and submucosal swelling, mainly localized in facial, abdominal, and/or extremities. In almost 80% of patients, it is inherited from an affected parent, and in the remaining cases results from a *de novo* event [1].

In type-1 HAE (~85% of cases), pathogenic variants cause a reduced esterase inhibitor protein synthesis. In type-2 HAE (~15% of cases) they cause the production of a non-functional protein, with an uncontrolled FXII/plasma kallikrein pathway activation and overproduction of the vasoactive peptide bradykinin, increasing the blood vessels permeability and resulting in tissue swelling and subsequent angioedema [1].

So far, more than 700 *SERPING1* pathogenic variants have been described as causative of C1-INH-HAE [1]. Single Nucleotide Variants (SNVs) represent the most frequent class, including missense variants (32%), splicing variants (14%), and nonsense variants (9%). Small indels account for 36% of identified pathogenic variants, while Copy Number Variants (CNVs) have been described in almost 8% of cases. Variants in regulatory regions (promoter, 5’-UTR, 3′-UTR) or deep intronic regions have been reported in a minority of cases (1%) [2].

In this case report, we aim to describe a very rare case of a 55-year-old patient with an unusual cytogenomics finding, characterized by the *SERPING1* whole-gene deletion on chromosome 11 and an additional independent deletion on chromosome 17, involving the *PMP22* (OMIM *601097) gene, associated with Neuropathy, Hereditary, With Liability To Pressure Palsies (HNPP, OMIM #162500) disease.

## Clinical description

A 55-year-old woman was referred for genetic counseling after a clinical diagnosis of C1-INH-HAE, characterized by C1-INH blood levels of 11 mg/dl (n.v. 15–35 mg/dl) and C1-INH functional activity of 10% (n.v. 70–130). She reported symptoms onset at age 4, presenting with swelling of the lips and of the extremities. Symptoms worsened with age, with the onset of recurrent and debilitating episodes after menarche, that reached a frequency of 2 episodes per month after menopause.

At 44 years, she presented the first life-threatening attack characterized by glottis edema leading to Emergency Room access, with subsequent clinical diagnosis. She started the treatment with on-demand and prophylactic plasma-derived C1-INH.

She also presented keratoconjunctivitis sicca at the vital dye staining and a positive Schirmer test in both eyes. Her extra-glandular involvement manifested in recurrent seronegative polyarthritis and symptoms suggesting peripheral neuropathy. Assessment of autoantibodies documented positive ANA with a homogeneous pattern and a 1:160 titer, along with SS-A and SS-B positivity. A diagnosis of primary Sjögren syndrome was made, and therapy with artificial tears and hydroxychloroquine was started. In the last five years, she reported a relevant worsening of musculoskeletal pain, mainly back pain, muscle weakness and cramps together with sleep disturbances, leading to the premature termination of her housekeeping work.

Electromyography (EMG) identified bilateral median neuropathy at the wrist with bilateral carpal tunnel syndrome and axonal neuropathy at the lower limbs.

In the family pedigree, she was the second daughter of four siblings born to a non-consanguineous couple with an unremarkable family history and no first-degree relatives reporting analogous symptoms.

## Molecular analysis

Genomic DNA was isolated from 200 μl peripheral blood mononuclear cells using the EZ1 DNA Blood Kit on an EZ1 Advanced XL automatic extractor (QIAGEN GmbH, Germany), following the manufacturer’s instructions.

Next Generation Sequencing (NGS) analysis was performed with an on-demand Ion AmpliSeq™ targeted panel (Thermo Fisher Scientific) comprising all coding regions and exon/intron junctions (±20 bp) of the *SERPING1* gene (NM_000062.3). The library and template were generated from 15 ng of genomic DNA using an automated methodology on Ion Chef™ instrument, and the samples were sequenced on the Ion GeneStudio S5™ Prime system (Thermo Fisher Scientific). Target coverage with a minimum read depth of 100x was > 99%. Sequences were aligned against the human reference genome assembly GRCh38/hg38. BAM and VCF files were generated using the pre-installed plugin in the Torrent Suite™ program (Thermo Fisher Scientific). Variants were described following HGVS Nomenclature v21.0 [3], searched through the databases LOVD, ClinVar, HAEdb, DECIPHER, HGMD, gnomAD v4.1., and classified according to the ACMG/AMP criteria [4].

Multiple Ligation-dependent Probe Amplification (MLPA) was performed using 50 ng of genomic DNA with the SALSA MLPA Probemix P243–B1 SERPING1–F12 (MRC-Holland, The Netherlands), according to the manufacturer protocol. PCR products were separated on a SeqStudio 8 Flex Genetic Analyzer capillary electrophoresis system (Applied Biosystems, USA) and analyzed with the Coffalyser.Net software v.220513.1739 (MRC-Holland, The Netherlands).

Single nucleotide polymorphism (SNP) array analysis was performed on genomic DNA in accordance with the manufacturer’s instructions, using Infinium CytoSNP-850 K BeadChip (Illumina, USA). The iScan system (Illumina, USA) generated array scanning data, which were analyzed by the Blue Fuse™ Multi Software Edition 4.5 (release hg38). CNVs have been classified according to and the ACMG/ClinGen recommendations [5].

Fluorescence In Situ Hybridization (FISH) analysis was performed using customized oligo probes targeting *PMP22* gene at 17p12 (chr17:15,196,480–15,296,324) (GRCh38/hg38) and *ACACA* gene at 17q12 (chr17:37,084,992–37,406,811) (GRCh38/hg38) as control probe (Agilent Technologies, USA).

## Results

NGS analysis did not disclose pathogenic or likely pathogenic SNVs in the *SERPING1* gene’s coding exons and exon-intron boundaries.

**Fig. 1:**
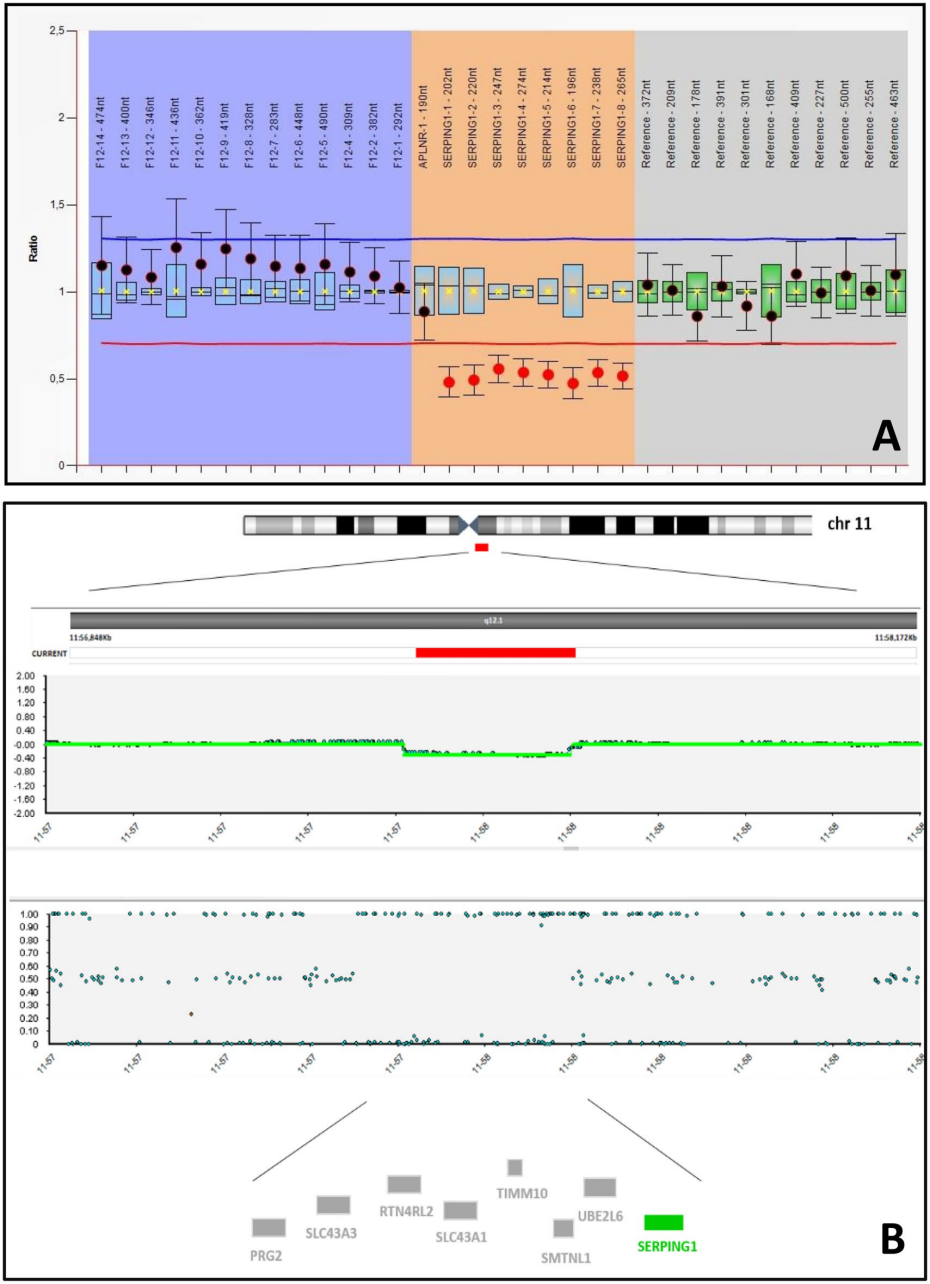
*SERPING1* deletion. (**A**) MLPA showing a heterozygous deletion of the whole *SERPING1* gene (red dots on orange background). (**B**) SNParray showing the heterozygous microdeletion of 251 kbp on chromosome 11q12.1 (red bar). Genes included in the deleted region are reported below: the disease causing *SERPING1* gene is shown in green, OMIM non-morbid genes are shown in gray

MLPA analysis unraveled the presence of the heterozygous variant NC_000011.10:g.(?_57597701)_(57614240_?)del (p.0?), corresponding to a chromosomic deletion involving the whole *SERPING1* gene (Fig. 1A).

To confirm this result and to identify breakpoints, a genome-wide chromosomal analysis was performed through SNParray, which disclosed a 11q12.1 microdeletion (arr[GRCh38] 11q12.1(57,389,932_57,640,909)x1) spanning almost 251 Kb and involving the whole *SERPING1* gene, confirming the clinical diagnosis of C1-INH-HAE. The deleted region does not involve other disease-causing genes (Fig. 1B) and it was not previously described in the literature or included in international databases (LOVD, ClinVar, HAEdb, HGMD, DECIPHER), representing a novel deletion. However, since *SERPING1* gene haploinsufficiency has been reported as causative of C1-INH-HAE, the deletion of the entire gene can be classified as pathogenic, according to ACMG/ClinGen recommendations (evidence: 2A) [5].

**Fig. 2:**
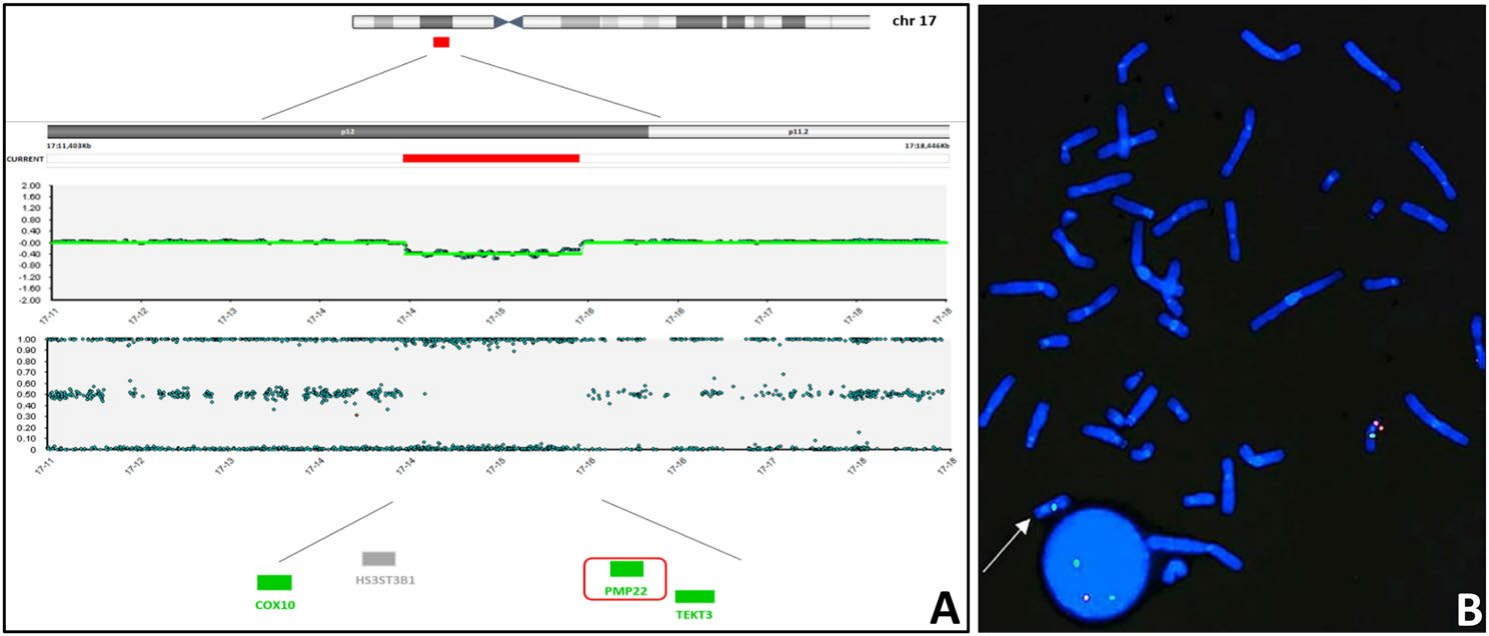
*PMP22* deletion. (**A**) SNParray showing the recurrent heterozygous microdeletion of 1.4 Mbp on chromosome 17p12 (red bar). Genes included in the deleted region are reported below: OMIM non-morbid gene is shown in gray, disease causing genes are shown in green; *PMP22*, the critical gene of 17p12 microdeletion syndrome, is highlighted in a red box. (**B**) FISH on lymphocyte metaphase spread of the patient using CMT1A-*PMP22* locus specific probe (red) with a *ACACA* (17q12) locus specific control probe (green), showing and confirming the heterozygous deletion of the CMT1A-*PMP22* locus (arrow)

This analysis also disclosed a 17p12 microdeletion (arr[GRCh38] 17p12 (14,184,151_15,567,865)x1), spanning almost 1.4 Mb (Fig. 2A), confirmed by FISH analysis (Fig. 2B), and involving the disease-causing gene *PMP22,* associated with HNPP disease. The deleted region involves two other disease-causing genes, *COX10* (OMIM *602125) and *TEKT3* (OMIM *612683) associated with autosomal recessive conditions (Mitochondrial complex IV deficiency, nuclear type 3 OMIM #619046 and Spermatogenic failure 81 OMIM #620277, respectively), which are not relevant for the clinical phenotype of the patient. The deleted region identified in our patient overlaps with the recurrent 17p12 microdeletion, known as pathogenic in the literature and international databases (LOVD, ClinVar, HGMD, DECIPHER).

## Discussion

C1-INH-HAE is a rare disease caused by *SERPING1* heterozygous pathogenic variants. Currently, HAE diagnosis is mainly based on C1-INH dosage and functional activity, C4 complement values, and clinical symptoms based on international consensus criteria [6].

The main clinical features may be shared with more common conditions (anaphylaxis, acquired angioedema) and accurate diagnosis may be missed or delayed, leading to unnecessary surgical procedures or ineffective treatments.

Available therapeutic approaches include on-demand and prophylactic treatments, based on the C1-INH replacement therapy, inhibition of bradykinin binding to its receptors, or reduction of plasminogen conversion and kallikrein activity [1]. Classical life-long therapeutic agents include plasma-derived C1-INH and monoclonal antibodies against plasma kallikrein [7].

Recent research has led to the development of targeted single-administration therapies, such as RNA-based treatments, gene editing, and gene therapy strategies for the definitive treatment of HAE [7, 8].

In this new scenario of precision medicine, genetic testing is crucial to confirm diagnosis and to open new opportunities for HAE individualized therapeutic approaches [8, 9].

We report the unusual case of *SERPING1* whole-gene deletion in a sporadic case of C1-INH-HAE. To date, a number of large deletions and large insertions/duplications in *SERPING1* have been described in patients with HAE [2, 10], mostly involving exons 4, 5, and 6 having a high recombination rate from *Alu* elements [11]. Besides this, the localization of *SERPING1* within a region near the centromere on chromosome 11 (Fig. 1) has been proposed as one of the causes of high genomic rearrangement rates in this gene [11].

To the best of our knowledge, few analogous cases of whole *SERPING1* gene deletion have been reported in literature, in both sporadic and familial cases [12–16], confirming that *SERPING1* is prone to mutagenic liability and marked mutagenic heterogeneity [12].

In addition, genome-wide microarray showed a second microdeletion, a recurrent CNV causing HNPP. Indeed, the 17p12 region is a well-known site prone to recombination [17]. Recurrent rearrangements are derived from non-allelic homologous recombination between low-copy repeats, explaining the similarity of the breakpoints between unrelated affected patients. The region contains the dosage-sensitive *PMP22* gene, coding for the Peripheral Myelin Protein 22. Duplication of *PMP22* is associated with Charcot-Marie-Tooth1A (OMIM #118220), while the reciprocal deletion is causative of HNPP.

HNPP is characterized by episodic and recurrent focal motor and sensory peripheral neuropathy with sensory and motor symptoms. In the present report, the proband suffered from progressive muscular weakness and pain and EMG showed signs compatible with the genetic unexpected finding, also disclosing carpal tunnel, a consequence of nerve involvement in HNPP neuropathy, one of the most underdiagnosed conditions in routine clinical practice.

In Medical Genetics, the co-occurrence of double SNVs causing different diseases is emerging as a more frequent event than previously thought, affecting up to 5% of individuals with a rare disease, and the resultant phenotypic complexity may present a challenge to physicians [18].

Conversely, in this report, we describe the unexpected co-occurrence of two different pathogenic CNVs, causing two different diseases with their respective clinical symptoms. It also documents an exceptional co-existence of a rare primary complement defect (C1-INH-HAE) with a complex connective tissue disease (Primary Sjögren syndrome) that is usually characterized by an acquired reduction of complement components related to immune complexes and autoimmunity [19, 20].

This case report confirms that accessibility to genetic testing is crucial for patients with HAE. In this scenario, applying high-throughput NGS technology coupled with MLPA and CMA allowed a timely and accurate molecular diagnosis of HAE.

Moreover, the unique genomic architecture of this case revealed the presence of two genetic disorders, leading to a dual genetic diagnosis at the age of 55 years for the proband and appropriate genetic counseling for family members, representing a unique opportunity in terms of diagnosis, treatment and understanding the genetic basis of rare undiagnosed diseases.

### Sitography (URLs)

https://www.omim.org

https://databases.lovd.nl/shared/genes

https://www.ncbi.nlm.nih.gov/clinvar/

http://hae.enzim.hu/

https://www.deciphergenomics.org/

https://www.hgmd.cf.ac.uk/ac/index.php

http://gnomad.broadinstitute.org

## Data Availability

All data will be available upon reasonable request at the corresponding author.

## Abbreviations

HAE: Hereditary angioedema
C1-INH: C1-esterase inhibitor
SNVs: Single Nucleotide Variants
CNVs: Copy Number Variants
HNPP: Neuropathy, Hereditary, With Liability To Pressure Palsies
EMG: Electromyography
NGS: Next Generation Sequencing
MLPA: Multiple Ligation-dependent Probe Amplification
SNP-array: Single nucleotide polymorphism
FISH: Fluorescence In Situ Hybridization
